# Applications of Computational Methods in Biomedical Breast Cancer Imaging Diagnostics: A Review

**DOI:** 10.3390/jimaging6100105

**Published:** 2020-10-08

**Authors:** Kehinde Aruleba, George Obaido, Blessing Ogbuokiri, Adewale Oluwaseun Fadaka, Ashwil Klein, Tayo Alex Adekiya, Raphael Taiwo Aruleba

**Affiliations:** 1 School of Computer Science and Applied Mathematics, University of the Witwatersrand, Johannesburg 2001, South Africa; arulebak@gmail.com (K.A.); rabeshi.george@gmail.com (G.O.); ogbuokiriblessing@gmail.com (B.O.); 2 Department of Biotechnology, Faculty of Natural Sciences, University of the Western Cape, Private Bag X17, Bellville, Cape Town 7535, South Africa; aklein@uwc.ac.za; 3 Department of Pharmacy and Pharmacology, School of Therapeutic Science, Faculty of Health Sciences, University of the Witwatersrand, Johannesburg, 7 York Road, Parktown 2193, South Africa; adekiyatalex@gmail.com; 4 Department of Molecular and Cell Biology, Faculty of Science, University of Cape Town, Cape Town 7701, South Africa

**Keywords:** cancer, breast cancer, diagnostics, imaging, computation, artificial intelligence

## Abstract

With the exponential increase in new cases coupled with an increased mortality rate, cancer has ranked as the second most prevalent cause of death in the world. Early detection is paramount for suitable diagnosis and effective treatment of different kinds of cancers, but this is limited to the accuracy and sensitivity of available diagnostic imaging methods. Breast cancer is the most widely diagnosed cancer among women across the globe with a high percentage of total cancer deaths requiring an intensive, accurate, and sensitive imaging approach. Indeed, it is treatable when detected at an early stage. Hence, the use of state of the art computational approaches has been proposed as a potential alternative approach for the design and development of novel diagnostic imaging methods for breast cancer. Thus, this review provides a concise overview of past and present conventional diagnostics approaches in breast cancer detection. Further, we gave an account of several computational models (machine learning, deep learning, and robotics), which have been developed and can serve as alternative techniques for breast cancer diagnostics imaging. This review will be helpful to academia, medical practitioners, and others for further study in this area to improve the biomedical breast cancer imaging diagnosis.

## 1. Introduction

Cancer is a non-communicable disease characterized by abnormal cell proliferation or cell division, with the ability to spread to other parts of the body [1]. Cancer continues to be a major public health problem and has been labeled as a global threat exacerbated by poor lifestyle choices and environmental factors [2,3]. Generally, cancer is classified according to the affected body part or tissue of origin. The most common cancer diseases include but are not limited to lung cancer, ovarian cancer, prostate cancer, head and neck cancer, breast cancer, etc. [4]. Indeed, breast cancer has been considered as one of the most common cancers diagnosed among women around the world. Breast cancer comprises 18% of the total cases of female cancer and approximately a million new cases are reported in the world every year [5]. Due to the ability of this type of cancer to metastasize to distant organs or lymph nodes, it has been considered to be the leading cause of mortality in females [5,6].

Due to the increase in the numbers of breast cancer over the years, there has been a rise in the number of computational models and algorithms for diagnosis and treatment to assist medical practitioners. A commonly, and frequently, used computational method is artificial intelligence (AI). Many AI related models have been developed for detecting and diagnosing diseases not only for breast cancer or mammography image analysis and classification [7] but for other diseases such as hycobacterium tuberculosis classification (MTC) [8], human immunodeficiency virus (HIV) therapy, screening, identification, and prediction [9], coronavirus disease 2019 (COVID-19) detection and diagnosis [10], etc. These AI models include machine learning, deep learning, and robotics. Rapid improvement in classification and learning algorithms is one of the main reasons these models have been widely used for these purposes with good and efficient results. Therefore, the contribution of this review is to provide a concise overview of past and present conventional diagnostics approaches in breast cancer detection and diagnosis. Further, we gave an account of several AI related computational models that have been developed and can serve as alternative models for breast cancer diagnosis. The remaining part of this paper is organized as follows: The types of biomedical imaging is presented in Section 2. The computational techniques used in breast cancer imaging diagnostics are outlined in Section 3. This section discusses the AI models used in breast cancer diagnosis. Finally, the paper is concluded in Section 4, where some points of future work are recommended.

## 2. Types of Biomedical Imaging

### 2.1. Mammography

Mammography is an excellent method used in primary breast imaging. It is used for early detection of abnormalities in the breast, especially those suspicious for breast cancer before it becomes apparent clinically, by using low-dose X-ray imaging to generate the images of the breast [11,12]. According to the United States of America preventive services task force (USPSTF), this type of breast imaging has been helpful in the earlier and better treatment for women over 40 years of age and has decreased breast cancer mortality by at least 30% [13]. Although this imaging approach remains the key for early breast cancer detection and screening, the overall accuracy of the test remains low and second-line accurate imaging techniques are required in some instances to lessen the number of unnecessary excisional biopsies [14,15].

Screening mammography is credited with the examination of an asymptomatic woman and decreases the risk of breast cancer-related death [16,17]. Conventional mammography has limitations in specificity and sensitivity, especially in dense breasts. The sensitivity of this type of imaging in breast cancer diagnostics is about 50 to 85%, depending on the density of the breast. Meanwhile, the sensitivity is below 50% in the dense breast due to tissue superposition; this is a major reason for the false-positive result, which leads to additional imagining and cost and false-negative results due to masking of true lesions [18,19,20].

In the breast, the normal internal mammary lymph node chain is usually below 5 mm in diameter. Metastases to this chain cannot be easily detected by mammography or ultrasonography clinical examination because they are normally covered by cartilaginous and bony structures of the chest wall [21,22]. The use of mammography in the detection of recurrent breast cancer is a challenging task due to changes in the architecture of the breast, mainly in fibrosis and scarring secondary to radiotherapy and surgery, resulting in difficulties to interpret mammograms. Breast compression is another major challenge faced by this modality due to accompanied pain which could lead to delayed diagnosis. Hence, considering all of the aforementioned mammography limitations, there is a call for alternative and more accurate methods that can resolve the imaging of dense breasts [19,20].

### 2.2. Tomosynthesis

Due to the limitations of mammography, breast tomosynthesis was introduced to the clinic because of its ability to produce three-dimensional information at a lower dose and its relative cost-effectiveness. Consequently, there has been an upsurge in interest in tomosynthesis. The Food and Drug Administration (FDA) has approved some products that are now in use and on the market [23]. This technique involves using X-ray projection images acquired over an arc to generate image slices for a partially 3D image [24]. Tomosynthesis allows for the generation of an arbitrary number of in-focus planes retrospectively from a series of radiograph projections obtained in a single motion of the X-ray tube [25]. Notably, a combination of tomosynthesis and digital mammography increases the brightness of invasive cancers while at the same time decreasing the likelihood of false-positive data [24]. Tomosynthesis has been applied to several clinical tasks, including dental imaging, angiography, breast imaging, bone imaging, and chest imaging [23]. In breast cancer, tomosynthesis increases the sensitivity of mammography, which could enhance the early detection of breast cancer due to the improved lesion margin conspicuity [25]. This is very beneficial to breast cancer patients, especially those with radiographically dense breasts. However, Poplack et al. [26] showed that breast tomosynthesis has a comparable or superior image when compared with diagnostic film-screen mammography in 89% of recruited subjects. More recently, this was supported by another study where one-view stand-alone digital breast tomosynthesis (DBT) detected more breast cancer than digital mammogram (DM) [27]. This suggests that the use of one-view DBT alone could be feasible in breast cancer screening. Although the acquisition procedures of tomosynthesis mimic standard mammography, the X-ray tube of tomosynthesis takes several low-dose exposures as it travels within a limited arc of motion unlike conventional mammography [26]. Sechopoulos [28] has written an excellent review of all aspects of tomosynthesis, including doses and reconstruction processes. When the overall dose used for visualization is constant, the quality of the image improves with a wider angular range [29]. However, the quality of image degenerates once the maximum is attained at a particular number of projections.

### 2.3. Ultrasound Imaging

Ultrasound (US) imaging diagnostics, otherwise known as sonography or ultrasound scanning, is a painless and safe approach. US makes use of 1 to 10 MHz sound waves to produce pictures that reveal the movement and structure of the breast, and other soft tissue [30,31]. It can also reveal the movements of blood and other materials within the blood vessels and body [31]. It is a cross-sectional technique that uses a small probe, known as a transducer, and gel that is directly placed on the breast/skin; it displays the tissues without overlap [31,32,33]. The high-frequency soundwaves travel from the probe via the gel into the body, and the probe receives the sounds that bounce back, which in turn produces an image on a computer. This type of imaging technique does not make use of radiation because it captures images in real-time [31,32,33].

In recent times, the development of high-resolution US technique has greatly improved the diagnosis of breast cancer because, in the past, US was thought to only be suitable for the diagnosis of cysts [34,35]. It has been shown to enhance the differential diagnosis of both benign and malignant lesions during guided interventional and local preoperative staging diagnosis. Due to the higher sensitivity of this type of imaging technique, it has been adopted as a complementary technique to mammography with limited sensitivity to identify early, node-negative cancer in dense breasts [36,37].

However, the use of US imaging techniques is diminishing due to the time and skill required to detect small tumors with hand-held imaging, and non-palpable cancers. The implementation of this imaging technique in breast cancer diagnostics has been hampered by limited numbers of qualified personnel and lack of uniformity in the results; this has caused low specificity that can lead to the generation of high numbers of false-positive results [38]. This assertion is corroborated by findings of some previous studies which revealed that US can identify and detect the presence of carcinoma in dense breasts. Some other studies have shown low detection of cancerous cells in dense breasts, but have proposed the addition of this imaging method to negate mammography which seems to have limited cost-efficiency and is controversial for women with dense breasts without any other major risk factors. In addition, due to the high scattering ability of the soundwaves at bone and air interfaces, various parts of the body are invisible, which limits the effectiveness of depth imaging in most organs to about 10 cm [39,40].

### 2.4. Dedicated Breast Computed Tomography

Dedicated breast computed tomography (DBCT) is a recently used and fastest-growing imaging technique that allows for true isotropic and provides three-dimensional (3D) information which can be reconstructed or rebuilt into several imaging planes. Although DBCT is comparable to breast magnetic resonance imaging (MRI), the process involved can be carried out without breast compression, and is not limited by breast implants or the density of the breast [41,42,43]. The radiation dose in this type of imaging technique is similar to that of a conventional two-view mammogram [42]. Boone et al. [44] investigated the feasibility of low dose radiation on the image quality of DBCT. The findings from their average glandular dose for 80-kVp breast CT study, when compared to two-view mammography, revealed that the breast CT dose for thicker breasts is approximately one-third lower than that of two-view mammography. For a typical breast of 5 cm 50% glandular, it was discovered that the maximum dose of mammography in 1 mm^3^ voxel is far greater (20.0 mGy) than that of breast CT with 5.4 mGy. It was further stated that the CT images for 8 cm cadaveric breasts have an average glandular dose of 6.32 mGy, which is superior to the estimated dose of 5.06 mGy for the craniocaudal view, with an average glandular dose of 10.1 mGy for standard two-view mammography of the same specimen [44]. The invention, improvement, and development of DBCT with dedicated scanners with novel technology has been documented in the literature by Sarno et al. [45]. Studies further reported the development of low radiation dose scanners with improved spatial resolution and rapid image acquisition times, which is aimed at addressing the issue of imaging dense breasts and painful breast compression [41,42,43].

Kuzmiak et al. [42] investigated the confidence of radiology experts in the characterization of suspicious breast lesions with a DBCT system compared with the conventional diagnostics of two-dimensional (2D) digital mammography in terms of overall lesion visibility and dose. It was discovered that DBCT is superior in the characterization of the masses and radiologists’ visualizations, although it is inferior to calcifications when diagnostic mammography is used. It was further averred that the DBCT application could help eliminate the 2D mammography drawback of overlapping tissue. Their study concluded that the technical challenges in breast imaging remain, but 3D DBCT could have a promising clinical application in breast cancer diagnosis or screening, however, this needs further investigation.

In 2008, Lindfors et al. [41] carried out a comparative study between the DBCT and screen-films mammograms where it was discovered, in the study of the selected group of women, that the visualization of breast lesions with both the DBCT and screen-film mammography is approximately the same. Although, DBCT was reported to be superior in the visualization of the masses, while in the imaging of microcalcification lesions screen-film mammography shows to be better. It was further deduced in their study that women are more comfortable with DBCT screening when compared to screen-film mammography. Hence, it was assumed that DBCT is a potential technology and may be a promising clinical application in diagnostic and screening for breast cancer investigation. Additionally, it was further presumed that DBCT is more accessible and could be a replacement for breast MRI or act as a control technique for tumor ablation procedures or robotic breast biopsy, all of this calls for further studies.

Recently, Shah et al. [43] investigated the characterization of computed tomography (CT). Hounsfield units were used in clinical settings for the purpose of tissue differentiation in a reconstructed CT image in 3D acquisition trajectories on a DBCT system. It was depicted in their statistical study that the approach has a better performance in the saddle orbit, mostly when close to the chest and the nipple areas of dense breast. It was further discovered that the saddle orbit functions significantly well and provides a tighter distribution of Hounsfield unit values in the reconstructed volumes. In addition, the study demonstrated the significance of the application of 3D acquisition for breast CT trajectories and other uses through the establishment of the robustness in Hounsfield unit values in the large reconstructed volumes.

### 2.5. Magnetic Resonance Imaging

Since the beginning of the third millennium, magnetic resonance imaging (MRI) has developed into a paramount tool in breast cancer screening, diagnosing, staging, and follow-up [46]. This imaging tool has played a vital role in the screening of high-risk breast cancer patients. Breast MRI uses an intravenous contrast agent such as gadolinium, which allows for the visualization of lesions. The sensitivity of this tool in breast cancer has been documented to be over 90% while the specificity is still about 72%; hence, the distinction between benign and malignant lesions is still challenging [46]. Although mammography is the basic imaging tool for breast tumor identification, it has been indicated that MRI has a higher sensitivity for detection of breast cancer, and the breast density does not affect it [47]. In most cases, the sensitivity of mammography in the detection of multiple malignant foci is below 50%. It is important to note that breast MRI is not meant to replace mammography particularly in ductal carcinoma in situ, which is not detectable by MRI but rather by mammography [48]. The MRI screening in women with genetic susceptibility to breast cancer has proved to be beneficial [49,50]. In a prospective cohort study, the sensitivity of MRI in women with a high risk of breast cancer but who were asymptomatic was between 93–100%, the 10-year survival was 95.3% [50]. Similarly, the sensitivity of MRI in contralateral breast tumor detection was documented to be 91%, and specificity was 88% [51]. In women with a known BRCA1/2 mutation, MRI surveillance detected breast cancer at early stages; encouragingly, there was no distant recurrence after 8.4 years follow-up since diagnosis [52]. This tool can be used in identifying the size and degree of the tumor towards achieving better surgery procedures. Nevertheless, the use of MRI before surgery continues to be controversial with extensive variations in the outcome; however, it helps in planning conservation in patients that respond to chemotherapy where feasible [46]. Despite the high sensitivity of this imaging tool in breast cancer, the cost involved in MRI makes it difficult to be employed in the general population. Conclusively, the invention and development of new imaging techniques such as diffusion-weighted imaging offer an added advantage in breast cancer management.

### 2.6. Diffusion-Weighted Imaging

Since the early years of the 21st century, diffusion-weighted imaging (DWI) has been at the forefront of cancer imaging attaining widespread recognition due to its ability in the diagnosis of stroke [53,54]. DWI is a noninvasive MRI technique that relies on the principle of random molecular motion of free water in tissues (Brownian movement). With the development of stronger diffusion gradients and application in whole-body imaging, DWI has attracted attention in oncology [55]. In breast cancer, Sinha et al. [56] demonstrated that DWI is reliable in a clinical setting with an echo-planar sequence and possesses potential in breast lesion characterization as either benign or malignant using apparent diffusion coefficient (ADC) values. Generally, breast lesions classified as malignant have a high-cellular level with limited water diffusion and lower ADC values when compared to benign lesions [57]. An earlier clinical study that recruited women with breast lesions stated that ADC values and the tumor biological aggressiveness correlate; hence, ADC is a promising factor in the evaluation and analysis of the degree of the malignancy [58]. In most clinical settings, DWI is interpreted in combination with dynamic contrast-enhanced (DCE)-MRI to increase the specificity. However, more recently, lesions in the breast (31 = malignant; 13 = benign) were analyzed using quantitative diffusion-weighted sequence on 3T MRI with b-values of 500 and 1000 s/mm^2^ [59]. The ADC cut-off value for benign and malignant lesions was set to 1.21 × 10^−3^ mm^2^/s for b = 500 s/mm^2^ and 1.22 × 10^−3^ mm^2^/s for b = 1000 s/mm^2^, respectively. The sensitivity of DCE-MRI was 100% with a specificity of 66.7%, when DCE-MRI was combined with b = 1000 s/mm^2^, 100% specificity was attained and sensitivity of 90.6%; there was no significant difference between the ADC and prognostic factors [59]. Non-contrast (NC)-MRI can be an alternative for DCE-MRI for breast cancer diagnosis, though its inferior lesion conspicuity and lower inter-reader agreement should be considered [60]. This study and many more have documented explanatory results for DWI as a tool for diagnosing breast lesion and aids the orthodox breast MRI procedures. Several pitfalls, which include but are not limited to motion artifacts, ADC value accuracy, image quality, and signal-to-noise ratio, are associated with DWI [61,62]. These challenges are bothersome and lay emphasis on the need to incorporate computer science into breast cancer diagnosis, for example, robotics could significantly decrease time in DWI MRI and create improved breast cancer detection.

### 2.7. Computed Tomography

CT scan is a method that exposes the pictures of cross-sections or 2D slices of the body’s organs via a connected computer [63,64]. The use of a contrast solution (iodine), injected into the body via the arm, dramatically improves and aids in the visualization of the cancerous cells in organs. In 2003, the use of CT for breast cancer imaging was proposed by Suga et al. [65], after a surgical issue in patients, to obtain interstitial lymphography that can map and present sentinel lymph nodes of the breast. The use of CT in breast cancer has some advantages, which includes patient comfort and fast scanning time. However, CT has not been widely used in breast cancers due to the risks involved in radiation exposure and poor quality of the image produced.

Due to the dynamic technique of CT, it can be used in the detection and characterization of breast tumors, investigation of neoadjuvant chemotherapy effects, and local staging of cancerous cells in the breast. In 2015, Foo et al. [66] employed this imaging scan method to evaluate the staging of cancer cells in newly diagnosed breast cancer patients that are in a locally advanced stage. It was revealed that a limited number of patients involved in this study had some pelvic significance with relation to a patient who had peritoneal cancer with widespread metastasis, and a patient with a presumed gene carrier of a concurrent primary ovarian malignancy. It was further stated that 50% of all pelvic results required additional radiological examinations.

Although the CT scan technique in breast cancer examinations may not replace the conventional mammography routine, based on improvements carried out in some studies [67,68], it can be used to overcome several limitations associated with mammography such as detection of cancers in premenopausal, dysplastic, and dense breasts. The mean glandular dose of 8.2 ± 1.2 mGy has also been documented for different types of breast shapes and sizes [69]. As documented by Park et al. [68], in prone positions, low-dose perfusion CT is possible for imaging with regards to the quantification of tumor vascularity and radiation doses. CT can be used in the detection of unsuspected very small cancers in the breast that cannot be identified or seen by physical examinations or conventional mammography. It is useful in definitive diagnostic evaluation in a situation where physical examinations and mammography are inconclusive, and it can also be helpful in recognition of precancerous and high-risk lesions. More so, CT can be used in the discrimination of tumor tissue from normal tissue in breast cancer patients without the use of a contrast medium.

### 2.8. Near-Infrared (NIR) Fluorescence

During human surgery, X-ray fluoroscopy and ultrasound have been used widely. However, during X-ray fluoroscopy, patients and caregivers are exposed to ionizing radiation; in an ultrasound, only a thin surgical field-of-view is seen and requires direct contact with tissue, in this case, breast. Interestingly, none of the methods can be amended by target contrast agents to guide imaging during oncologic surgery due to the number of procedures required [70,71]. Thus, near-infrared (NIR) light, with a wavelength range of about 700 to 900 nm, has offered diverse significant advantages over some widely used tools including relatively high penetration of photon in and out of living tissue (breast) due to the reduction in the rate of absorbance and scatter. Owing to lower tissue autofluorescence, NIR has a higher signal-to-background ratio [71,72]. This technique has a great potential to interrogate deep tissues (breast) for molecular-based imaging. The NIR light is visible to the human eyes when conjugated with NIR excitable fluorophore or dyes. These are chemical compounds which convert light generated from one NIR wavelength into the NIR light of diverse wavelength. It has been recommended that the mapping of sentinel lymph nodes (SLN) is a standard approach for the management of breast cancer and care staging of the axilla [71].

NIR fluorescence imaging, which uses indocyanine green (ICG), has been shown to improve the procedure of the SLN mapping by facilitating percutaneous incisions and identifying the intraoperative ability of lymphatic channels and SLNs [71,72]. The safety and accuracy of NIR fluorescence imaging applications for identifying SLNs in patients suffering from breast cancer were demonstrated by Verbeek et al. [73]. The use of the Mini-FLARE camera system and 1.6 mL of 0.5 mM ICG showed the excellent identification of the SLN in patients with breast cancer. Although, the technique which should be used as the gold standard in future analyses, was raised as a question [73]. In a similar study by Mieog et al. [74], the clinical translation of a novel NIR fluorescence imaging system and the optimal ratio of ICG to the human serum albumin (HSA) dose for mapping of SLN in breast cancer was described. It was stated that 400 and 800 μM is the optimal dose of the injection ratio of ICG:HSA and this can be chosen based on the preferences of local preparation. For instance, a dose of 500 μM was depicted to be the most convenient in the United States due to the minimal requirement in the manipulation of albumin volumes. Other studies that have employed this approach in mapping SLNs in breast cancer patients include Sevick-Muraca et al. [75] which demonstrated the prospective feasibility in the use of the minimal dose of ICG in noninvasive optical imaging of lymph nodes in the breast cancer patients undergoing SLNs mapping. In 2008, Altınoǧlu et al. [76]. demonstrated the synthesis and bioresorbable use of calcium phosphate nanoparticles (CPNPs) which incorporated the molecule of the NIR emitted fluorophore and ICG. In their study, the in vivo and ex vivo studies demonstrated the potentiality of the NIR CPNPs in diagnostic imaging of early breast solid tumors. Although, the result from their ex situ imaging of deep tissue showed that the depths of NIR CPNPs in porcine muscle tissue is 3 cm. Poellinger et al. [77] employed the use of NIR fluorescence imaging with the late and early enhancement of ICG, which corresponds to extravascular and vascular phases of contrast agent enhancement to distinguish between malignant and benign breast lesions as well as to detect breast cancer. Ke et al. [78] assessed the specificity of continuous-wave NIR fluorescence imaging by an intensified charge-coupled device (CDD) camera on a novel epidermal growth factor (EGF)-Cy5.5 to detect EGF receptors in breast cancer xenografts.

### 2.9. Single-Photon Emission Computed Tomography

Single-photon emission computed tomography (SPECT) is a medical imaging tool based on tomographic reconstruction protocols and routinely used in a clinical decision in cancer [79], coronary artery disease, left ventricular dysfunction [80], and Parkinson disease [81]. In fact, it is the most used tool in myocardial ischemia assessment. SPECT aims at getting a perfect 3D radioactivity distribution resulting from the uptake of a radiotracer in humans. One or more photons are released in random directions when a SPECT radioisotope decays [82]. However, collimators are used to focus the angle of the emitted photons that reach the detector because conventional lenses cannot restrict high-energy photons, and only 0.02% of the decay events is measured [82]. SPECT, coupled with CT, can be used when conventional images are complex to interpret, for example, suspicion of contamination [83]. Clinically, SPECT/CT provides more value in anatomical localization of sentinel nodes. This highlights a relevant role for this tool in the surgical approach and may improve staging [84]. The sentinel lymph node biopsy is a well-known procedure used in evaluating the status of the axillary lymph node in patients with early stages of breast cancer [85]. Markedly, SPECT/CT improved visualization from 84% to 92% in patients, but it only showed sentinel nodes in 11 out of 22 breast cancer patients (50%) with non-visualization on planar imaging [84]. Similarly, Lerman et al. [86] documented that the addition of SPECT/CT to lymphoscintigraphy enhances sentinel node identification in breast cancer patients who are overweight. Notably, SPECT/CT identified hot nodes in 91% of patients and sentinel nodes in 29 of 49 patients (59%) who were negative on planar imaging (planar lymphoscintigraphy) [86]. Hence, this technique is of high relevance in overweight breast cancer patients because intraoperative techniques have failed in the identification of draining nodes. Another SPECT/CT evaluation study demonstrated a sentinel node in 91.1% of breast cancer patients, and localization was more precise on SPECT/CT fusion images than on the planar views [87]. Mann et al. [88] documented that the use of dedicated SPECT identifies regions of interest at a global lower-level threshold within dense breast tissue without any negative effects, which in turn betters patient care. Additionally, dedicated breast positron emission tomography (PET)/CT can accurately visualize uncompressed breast suspected lesions in 3D [89]. However, this scanner was unable to generate a full quantitative image. Recently, Tornai et al. [90] developed a fully 3D CT in a hybrid SPECT/CT breast imaging system that facilitated complex trajectories, which improved the quality of the image when compared with simple circular breast CT acquisitions. The SPECT-subsystem allows viewing of the chest wall for pendant breast imaging [90]. Recently, it was shown that the hybrid SPECT/CT provides precise anatomical data that enables clear assessment of patients contaminated with radionuclide during the procedure [83]. Such precise data can assist surgeons towards a better surgical plan. Non-visualization of sentinel nodes, unexpected lymphatic drainage, and complicated planar imaging interpretation are challenges faced by these imaging techniques. However, this can be amended by incorporating AI, such as deep learning and machine learning algorithms, with currently available breast cancer imaging tools. Overall, such combinations will improve breast cancer diagnosis, predict treatment outcome and ultimately, improve the patient quality of life. The dose in the dedicated SPECT-CT system using both the geometric and anthropomorphic phantoms showed that the average doses absorbed in 100% fibroglandular-equivalent was 4.5 ± 0.4 mGy, while 100% adipose-equivalent tissues was 3.8 ± 0.2 mGy. More so, the dose measured in a cadaver breast using a radiochromic film in the same study yielded an average dose of 4.3 ± 0.3 mGY and 4.2 ± 0.3 mGy along two orthogonal planes [91].

## 3. Computational Techniques Used in Breast Cancer Imaging Diagnostics

A correct diagnosis of mammograms containing malignant tumors is a complex task for even the most experienced medical practitioner. To circumvent this complexity, several computational models have been developed to assist medical practitioners to distinguish between benign and malignant breast tumors. The models described in this paper are based on machine learning, deep learning, and robotics which have been shown to be useful in breast cancer diagnosis. In this section, we present some studies that have applied these models.

### 3.1. Machine Learning Algorithms

Several machine learning algorithms have been proposed for the detection and diagnosis of breast cancer. Despite this, the development of new algorithms and models for this purpose is still an active research area, especially in the detection of abnormalities in mammograms. In the following, we review machine learning models that have been used in diagnosing this type of cancer, such as artificial neural network (ANN) and support vector machine (SVM).

#### 3.1.1. Support Vector Machines

SVMs are supervised learning models that aim at formulating a computationally effective approach of learning to separate hyperplanes in high-dimensional feature space [92]. It has been used and proven to be an efficient learning technique for several real-world problems such as image recognition [93], bioinformatics [94], and classification problems [95], among others. SVMs are one of the earliest machine learning techniques used for cancer diagnosis. Acharya et al. [96] focused on detecting breast abnormalities or cancer automatically by using infrared imaging. The approach used texture features and SVMs to detect breast cancer based on thermography. Texture features were obtained from a run-length matrix and co-occurrence matrix from 25 cancerous and 25 normal infra-red breast images. These features were then fed to an SVM for automatic classification and detection of malignant and normal breast conditions. A comparison of SVMs based classifiers with ANNs and Bayesian classifiers for the prognosis and diagnosis of breast cancer was done in Maglogiannis et al. [97]. The implementation of the comparison was performed on the Wisconsin prognostic breast cancer and the Wisconsin diagnostic breast cancer datasets. The expected result of the implementation was to predict a class that corresponds to a likely tumor recurrence in four-time intervals. The result also shows that SVM outperforms the other classifiers. Huang et al. [98] used SVM to evaluate several pathologically proven breast tumors. The study presented a computer-aided diagnosis (CAD) system with textural features for classifying malignant and benign breast tumors on medical ultrasound systems. The aim of the CAD is to assist medical practitioners and radiologists in identifying lesions and also to differentiate malignant lesions from benign lesions on the basis of medical images. The proposed SVM technique was able to identify solid breast nodules at very high accuracy. Recently, Wang et al. [7] proposed an approach to solving the limitations of machine learning models’ performance in diagnosing breast cancer. The approach was based on an SVM-based ensemble learning algorithm; this approach reduces the diagnosis variance and increases diagnosis accuracy. In doing this, 12 different SVMs were hybrid using the proposed weight area under the receiver operating characteristics curve ensemble (WAUCE) approach.

#### 3.1.2. Artificial Neural Network

**Figure 1:**
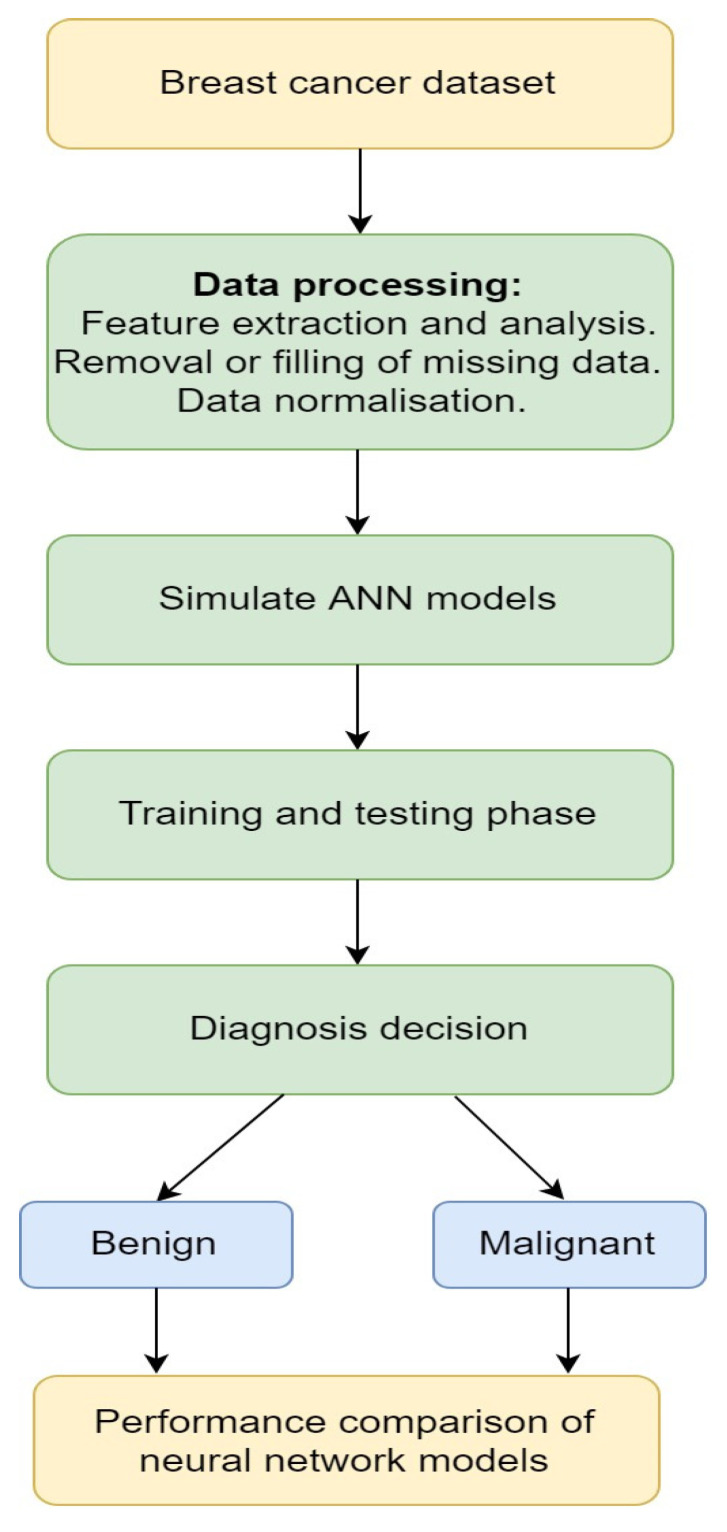
A simple artificial neural network (ANN) method on breast cancer [103].

ANN is a computational-intelligent model that uses different optimization tools to learn from the data available in the past and use that prior training to identify or predict new patterns or to classify new data. Several research works have applied ANN for medical purposes [99], such as cancer treatments [100]. The Memetic Pareto ANN (MPANN) approach was proposed by Abbass [101]. The approach was based on a pareto-differential evolution algorithm. This algorithm was augmented with a local search for the prediction and diagnosis of breast cancer. Tourassi et al. [102] proposed a new approach for breast cancer diagnosis based on the constraint satisfaction neural network (CSNN) technique using mammographic and breast cancer patient history findings. The main advantage of this technique is that it has a non-hierarchical architecture and flexibility that allows it to work as a predictive tool and as an analysis or data mining tool to discover the knowledge of association rules among clinical diagnosis and historical findings. In this work, the authors used two different datasets of breast cancer, each containing 250 patient cases. The CSNN was first used to train the first 250 datasets and the other 250 datasets were used to test the predictive strength of the CSNN. The result of the analysis was done based on the kind of mammographic lesions seen in each patient. The result of this study shows that CSNN is a very efficient CAD tool for predicting and diagnosing breast cancer from mammographic and historical findings. A study by Janghel et al. [103] implemented a model using ANN to assist medical practitioners in diagnosing breast cancer. The model has four phases, namely radial basis function networks (RBFN), back propagation algorithm (BPA), competitive learning network (CLN), and learning vector quantization (LVQ). The dataset used in this study consisted of 55 malignant cases and 184 benign cases. The result of the experiment showed that the LVQ output was the best result during testing then CLN, BPA, and RBFN in order. Figure 1 presents a simple ANN diagnosis for breast cancer.

Other works in the literature used data mining methods in diagnosing breast cancer [104,105]. Data mining is the process of extracting useful data from a larger set of raw data using one or more software. Çakır et al. [106] used Weka, a data mining tool to analyze 462 breast cancer patients data obtained from the Ankara Oncology Hospital. Classification algorithms are applied to each of the datasets and the outputs of the classification were compared to find the most effective treatment method. This work assists oncology doctors to suggest the best treatment method for a patient. Şahan et al. [107] proposed a hybrid system of a fuzzy-artificial immune system with the k-nearest neighbor (KNN) algorithm. This method was used to solve diagnosis problems through classifying the Wisconsin breast cancer dataset (WBCD). The system has a high classification accuracy on large datasets and can be used for any type of breast cancer diagnosis. Additionally, it can be used for other medical condition diagnoses. The Table 1 below presents an overview of machine learning (ML) techniques in breast cancer diagnosis that are explained in this section. The evaluation results presented in the table are the 50–50% training–test partition for the reference with three different training-test partitions.

### 3.2. Deep Learning

In recent years, deep learning has set an exciting trend in the fields of machine learning and AI [113]. Deep learning techniques utilize computational models, composed of multiple processing layers that are used to learn data representations and applied to many real-world applications. These applications range from image recognition, object detection, power systems, breast cancer detection, speech recognition to drug discovery and genomics, etc. [114,115,116,117]. In the following sections, deep learning models for breast cancer diagnosis are presented.

#### 3.2.1. Convolutional Neural Network

**Figure 2:**
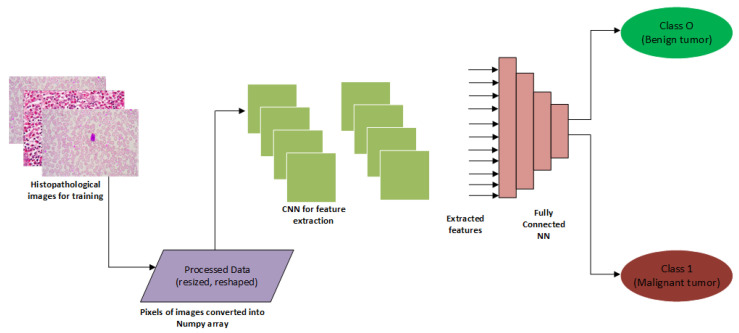
A convolutional neural network (CNN)-based approach for screening mammography [125].

The convolutional neural network (CNN), often called ConvNet, is a type of deep learning model that has become dominant for many computer vision tasks, ranging from image classification, object tracking and detection to semantic segmentation [118,119]. CNN was designed to adaptively learn hierarchies of features, usually from low-level to high-level patterns [120]. Indolia et al. [121] explained that the CNN overcomes limitations as seen in traditional machine learning approaches; it has shown to be widely used for solving complex problems, especially in the medical imaging domain. Recent studies have adopted the CNN model for effective breast cancer diagnosis [122,123,124]. An example of CNN segmentation tasks for breast cancer diagnosis is presented in Figure 2.

For an improved diagnosis, Tan et al. [126] developed an imaging system called breast cancer detection using convolutional neural networks (BCDCNN) aimed at assisting medical practitioners to classify mammographic images into malignant or benign. The results showed that the BCDCNN system improved the accuracy of the classification tasks on the mini-Mammographic Image Analysis Society (mini-MIAS) database. Amit et al. [122] proposed an approach for dynamic contrast-enhanced (DCE) imaging that uses the CNN to correctly classify medical images and a pre-trained classifier to extract features in the images. The study showed that CNN outperformed the pre-trained classifier and accuracy improved significantly. In another study, Byra et al. [127] described a CAD approach that uses the Nakagami imaging method to train a CNN model, aimed at breast cancer diagnosis. The study was tested on 458 RF data matrices of breast lesions. The study showed that better area under the curve (AUC) results that amounts to 0.912 were obtained. Gao et al. [128] extended the use of CNN using the INbreast dataset to overcome the challenges faced with the contrast-enhanced digital mammography (CEDM), which is prone to a high false-positive rate. CNN was effective at differentiating benign cases from malignant lesions, which is the main challenge faced with a breast cancer diagnosis.

In a similar study, Wang et al. [129] explored a CAD method that utilizes feature fusion with CNN using a private dataset. The method uses CNN for feature extraction based on several image sub-regions. After the feature extraction tasks, the images were then classified into benign or malignant. The study concluded that this task outperformed other existing methods. Murtaza et al. [124] applied the use of CNN on the BreakHis dataset to improve the detection of breast cancer. The study reported a high accuracy with the use of the CNN model. Other interesting areas of application of CNN to breast cancer diagnosis are found in the following references [126,130,131,132,133,134,135,136].

#### 3.2.2. Generative Adversarial Networks

**Figure 3:**
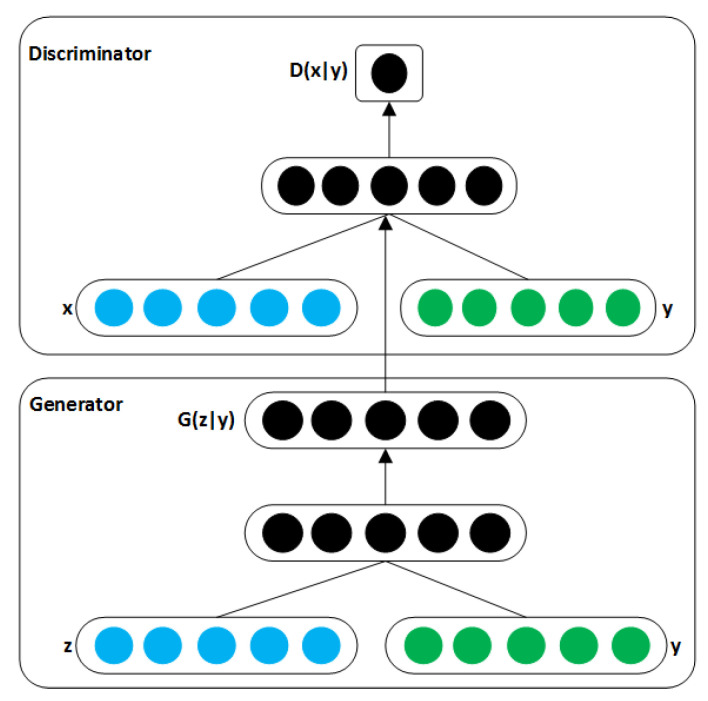
Structure of a generative adversarial networks (GANs) [142].

The advent of generative adversarial networks (GANs) by Goodfellow [137] has opened a new area of research within the image segmentation domain. According to Kazeminia et al. [138], GANs have been shown to generate realistic-looking images in the large, unlabelled corpus. One of the many challenges faced within the CV and medical image analysis (MIA) community is the heavy reliance on labelled training data, which can be a major limitation [7]. The communities have recognized the potential of GANs and have eagerly investigated in its efficacy to tackle many problems. Recently, a good deal of research has leveraged the use of GANs for image-to-image translation [139,140]. GANs have found many applications in generative modelling and distribution learning [139]. Furthermore, GANs unique generation and identification network is increasingly used for image segmentation and has achieved good results. GANs create outputs using its discriminator and generator [141]. Figure 3 shows the structure of GANs.

Shams et al. [143] developed DiaGRAM (deep GenerRAtive multi-task), which is based on GANs and CNN in a mammography study to detect early signs of breast cancer. The study concluded that feature learning with GANs led to high classification performance and an effective end-to-end scheme. A study by Singh et al. [144] applied GANs to segment mammographic images from regions of interests (ROIs) with varying length and sizes. GANs helped eliminate issues of overfitting on the datasets (INbreast and digital database for screening mammography (DDSM)) and showed effectiveness in the screening of cases. Wu et al. [145] addressed the issue of limited data and class imbalance for breast cancer classification using GANs. The classification performance of GANs was compared with other augmentation methods. The results showed that GANs improved the classification task. Guan et al. [146] applied GANs to generate synthetic images from a digital database for screening mammography. The authors opined that GANs performed better in augmenting the training dataset, which was useful for the study. Together, we have discussed the CNN and GANs approaches to breast cancer detection. Most of the works presented in this section are summarised in Table 2. In the table, we present the deep learning techniques, and scope of work that are used for breast cancer diagnosis. In addition, the performance metrics for each technique and the type of dataset used were presented.

### 3.3. Robotics

With the improvements in medical robots’ accuracy, robots in healthcare now assist by relieving medical practitioners from their routine tasks and also make medical procedures less costly and safer for patients [129]. These could be the reasons research into creating robots to detect and diagnose breast cancer in patients have been gaining popularity in the last decade. Robotics as a branch of AI, is developed based on some machine learning algorithms [129,150]. Such algorithms include but are not limited to reinforcement learning and deep reinforcement learning [150].

#### 3.3.1. Reinforcement Learning

Reinforcement learning is an approach to machine learning that involves computational learning by interaction. It involves learning about what to do and how to map situations to actions to maximize a numerical solution. Unlike other machine learning approaches, reinforcement learning does not directly demonstrate how to perform a task but works through the problem on its own [129,150].

Examples of systems that are built based on the unsupervised learning approach of reinforcement learning are self-driving cars, a program playing chess (e.g., Alphago), etc. These systems interact with their environment, such that, when they complete a task successfully, they receive a reward state. Such tasks could be driving to a destination safely or winning a game. On the other hand, when the system does not complete a task successfully, they receive a penalty for performing incorrectly. Such a task could also be going off the road or being checkmated [150].

These systems, over time, make decisions to maximize their reward and minimize their penalty using dynamic programming. The advantage of this approach to AI is that it allows an AI program to learn without a programmer spelling out how a system should perform the task; this type of approach is popularly called unsupervised learning [150].

#### 3.3.2. Robotic Tools for Breast Cancer Diagnosis

In the research reported by Kato et al. [151], a robotic system called WAPRO-4 was developed for the automatic palpation of breast cancer. The study aimed at palpating and diagnosing breast cancer without the assistance of medical personnel. The major objective was to aid the early detection of breast cancer. Additionally, WAPRO-4 consists of three parts which include the measuring instrument, the locomotion unit, and the microcomputer system [151]. The WAPRO-4 was constructed to detect tumors while ignoring breathing and the configuration of the chest wall. Kobayashi et al. [152] developed a palpation-based needle insertion method for diagnostic biopsy and treatment of breast cancer. The system locates cancerous tissues from force information and reduces tissue during needle insertion. Kobayashi et al. [152] compared the palpation-based needle insertion approach to the normal needle insertion approach using a numerical simulation of a breast tissue model. The outcome showed that palpation-based needle insertion had a smaller error which suggests that the procedure could be a safe and effective alternative [152].

Larson et al. [153] developed a robotic device to perform minimally invasive breast interventions with real-time MRI guidance for the early diagnosis and treatment of breast cancer. In this work, five computer-controlled degrees of freedom were used to perform minimally invasive interventions inside a closed MRI scanner. According to Larson et al. [153], the intervention consists of a biopsy of the suspicious lesion for diagnosis, which involves the therapies to destroy or remove malignant tissue in the breast. As a result, the procedure includes conditioning of the breast along with a prescribed orientation, the definition of an insertion vector by its height and pitch angle, and insertion into the breast. The entire device is made of materials compatible with an MRI machine, avoiding artefacts and distortion of the local magnetic field. This device was built to be remotely controlled via a graphical user interface.

Meanwhile, automated detection of breast lesions from DCE-MRI volumes was implemented based on deep reinforcement learning [154]. The method significantly reduces the inference time for lesion detection compared to an exhaustive search, while retaining state-of-the-art accuracy. The authors demonstrated their results on a dataset containing 117 DCE-MRI volumes, validating runtime and accuracy of lesion detection [154,155].

Moreover, Tsekos et al. [155] implemented a robotic device with haptic, tactile, and ultrasound capabilities, that can acquire and render the information of breast pathology remotely. In this work, the device is designed to screen for breast cancer by examination for the patient in a remote area without convenient access to medical personnel. The device was said to be more accurate than human medical personnel [155]. Further, a robotic based device designed to assist medical personnel in placing the instrument on the tumor location and automatically acquiring tumor images in real-time was implemented in [153,156]. This device has the potential to increase targeting accuracy while reducing the level of skill required to perform minimally invasive breast interventional procedures.

## 4. Conclusions

Breast cancer has shown to be one of the leading causes of female mortality in the world. Recent studies have shown that early diagnosis is the first step towards a successful treatment, which can help save many lives. This review presented a brief overview of past and present conventional diagnostics approaches as well as recent computational techniques that have contributed immensely to the diagnosis of breast cancer. Articles on breast cancer classification published from 2006 to 2020 were extensively reviewed. In total, 55 were carefully reviewed from different academic repositories. Several criteria were used for the review, such as conventional diagnostics approaches, the computational technique used, scope, evaluation results, and different types of datasets were used for these studies. We noticed that researchers preferred publicly available datasets over exclusive ones. For example, WBC and DDSM were seen to be popular among researchers. For computational approaches, we reviewed three areas: Machine learning, deep learning, and robotics. Out of these approaches, the deep learning techniques appear to be increasingly popular for most researchers. Among these techniques, we noticed that CNN was a popular choice, used for classification. Currently, GANs have shown to be a promising deep learning algorithm for breast cancer diagnosis due to its ability to give convincingly good results. Performance metrics such as AUC, accuracy, sensitivity, specificity, and measure were used for evaluating deep learning approaches.

Finally, this review provides a roadmap for future conversations about building better techniques for early detection, which could help save millions of lives. We believe that this comprehensive review will offer a better understanding of the breast cancer classification domain and provide valuable insights to researchers in this field.

## Figures and Tables

**Table 1:** An overview of machine learning (ML) techniques in breast cancer diagnosis.

Reference	Computation Technique	Scope	Evaluation Results	Datasets
Acharya et al. [96]	Texture features + SVM	Breast cancer detection using thermal imaging	Accuracy = 88.10%, specificity = 90.48%, sensitivity = 85.71%	25 normal and 25 cancerous collected from Singapore General Hospital, Singapore
Maglogiannis et al. [97]	SVM	Diagnosis and prognosis	Accuracy = 96.91%, specificity = 97.67%, Sensitivity = 97.84%	Wisconsin prognostic breast cancer (WPBC)
Huang et al. [98]	SVM	Classifying benign and malignant	Accuracy = 94.4%, specificity = 94.4%, Sensitivity = 94.3%	250 images of benign breast tumors from 215 patients and carcinomas from 35 patients.
Wang et al. [7]	SVM	Reduce the diagnosis variance and increase the diagnostic accuracy of breast cancer	Variance = 97.89%, increase in accuracy by 33.34%	Wisconsin Breast Cancer, Wisconsin Diagnostic Breast Cancer, and the U.S. National Cancer Institute’s Surveillance, Epidemiology, and End Results (SEER) program
Abbass [101]	EANN	Diagnosis	Average accuracy = 0.981 ± 0.005	Wisconsin
Bhardwaj et al. [108]	Genetically optimized neural network	Classification	Accuracy of 98.24%, 99.63% and 100% for 50–50, 60–40, 70–30 training–testing partition, respectively	WBCD
Tourassi et al. [102]	CSNN	Diagnosis	CSNN ROC area index = 0.84 ± 0.02	500 private images
Çakır et al. [106]	Weka	Treatment methods	Accuracy = 92%	462 patients data
Karabatak [109]	Weighted Naïve Bayesian	Detection	Sensitivity = 99.11%, specificity = 98.25%, accuracy = 98.54%	WBCD
Şahan et al. [107]	Fuzzy + KNN	Diagnosis	Accuracy = 99.14%	WBCD
Bagui et al. [110]	Rank nearest neighbor	Diagnosis	Accuracy = 98.1%	WBCD
Chen et al. [111]	Rough set_SVM	Distinguishing benign breast tumour from malignant one	Accuracy = 99.41%, Sensitivity = 100%, specificity = 100%	WBCD
Polat et al. [112]	Least square SVM	Classification	Accuracy = 94.87%, Sensitivity = 96.42%, specificity = 95.86%	WBCD

**Table 2:** Summary of deep learning models in breast cancer diagnosis.

Reference	Deep Learning Technique	Scope	Evaluation Results	Datasets
Tan et al. [126]	CNN	Classification	Accuracy = 82%	mini-Mammographic Image Analysis Society (mini-MIAS)
Amit et al. [122]	CNN	Classification	Accuracy = 83%, Area under the curve = 0.91	ED (MRI)
Byra et al. [127]	CNN	Classification	Accuracy = 83%, Area under the curve = 0.912	ED (US, Nakagami)
Gao et al. [128]	CNN	Classification	Accuracy = 90% Area under the curve = 0.92	INbreast
Wang et al. [129]	CNN	Classification	Accuracy = 76.5%	Private
Tan et al. [126]	CNN	Classification	Accuracy = 95%, Area under the curve = 0.97	BreakHis
Litjens et al. [130]	CNN	Classification	Area under the curve = 0.99	Private
Araújo et al. [132]	CNN	Classification	Accuracy = 77.8% (four classes), Accuracy = 83.3% (two classes)	BICBH
Ragab et al. [135]	CNN with SVM	Feature extraction	Accuracy = 73%, Area under the curve = 0.94	Digital Database for Screening Mammography (DDSM), CBIS-DDSM
Acharya et al. [136]	CNN with K-means	Feature extraction	Accuracy = 97%	Private
Karthik et al. [147]	DNN	Classification	Accuracy = 98%	WBC
Yu et al. [134]	DNN + CNN	Classification	Accuracy = 81%, Area under the curve = 0.88	BCDR
Sun et al. [131]	CNN	Classification	Accuracy = 82.43%, Area under the curve = 0.8818	ED(Mg)
Hadad et al. [148]	CNN	Classification	Accuracy = 94%, Area under the curve = 0.98	ED(Mg, MRI)
Nahid et al. [123]	CNN	Classification	Accuracy = 91%	BreakHis
Shams et al. [143]	GANs	Classification	Area under the curve = 0.88, Area under the curve = 0.925	DDSM, INbreast
Singh et al. [144]	GANs + CNN	Classification	Accuracy = 72%	DDSM and Private
Wu et al. [149]	GANs	Classification	Accuracy = 89%	DDSM
Guan et al. [146]	GANs	Classification	Accuracy = 79.8%	DDSM
